# The psychosocial impacts of skin-neglected tropical diseases (SNTDs) as perceived by the affected persons: A systematic review

**DOI:** 10.1371/journal.pntd.0012391

**Published:** 2024-08-02

**Authors:** Dasha L. Alderton, Caroline Ackley, Mei L. Trueba

**Affiliations:** Brighton and Sussex Medical School (BSMS), University of Sussex, Brighton, United Kingdom; KARI-Trypanosomiasis Res Centre, KENYA

## Abstract

**Background:**

Neglected Tropical Diseases (NTDs) disproportionately affect marginalised groups within impoverished communities, conferring devastating physical, financial and psychosocial effects. Skin-NTDs (SNTDs) are uniquely stigmatising due to their visible nature, rendering affected individuals vulnerable to psychosocial risk and the associated decline in social participation, quality of life and mental health. In response to knowledge gaps identified by current global efforts for integrated control of SNTDs this review gathers existing evidence on the psychosocial effects of SNTDs, with consideration given to the influence of gender.

**Methods:**

The study protocol is registered with PROSPERO (CRD42022336676). Data was collected from Embase, Global Health, Medline and Web of Science, with additional articles identified through Google Scholar and bibliography tracking. Qualitative studies published in English between 2005 and 2024 reporting men’s and women’s experiences with SNTDs were searched. Appropriate data from each included study were inputted into NVivo software to facilitate thematic synthesis. Descriptive and analytic themes were generated through line-by-line coding using an inductive approach.

**Results:**

27 articles of high and moderate quality were included. They pertained to buruli ulcer, cutaneous leishmaniasis, leprosy, lymphatic filariasis, tungiasis, onchocerciasis, schistosomiasis and podoconiosis. Men and women across SNTDs and contexts reported debilitating physical symptoms which impaired their ability to work, socialise and carry out usual daily activities. Some felt (at least initially) well supported by partners and relatives, whereas most experienced avoidance, abandonment and even violence, with women incurring worse SNTD-related social consequences. Many men and most women experienced stigma, with discriminatory behaviours largely attributed to fear of infection, decreased ability to perform gender-specific daily activities, and the perceived association between SNTDs and sinfulness. Self-reported impacts of SNTDs on men’s and women’s mental wellbeing included low mood, anxiety, low self-esteem, and suicidal ideation. Disease-specific knowledge, early treatment, social support, and disease acceptance were mentioned as protective factors.

**Conclusion:**

SNTDs cause significant psychosocial harms, particularly for women. Implementing myth-busting and contact-based educational campaigns and improving access to treatment and to livelihood opportunities and social protection schemes for men and women with a SNTD will help prevent and mitigate these.

## Author summary

Neglected Tropical Diseases (NTDs) mostly affect marginalised groups in deprived communities, and are known to have harmful physical, financial, social, and mental health consequences. Skin-NTDS (SNTDs) are particularly damaging because they are noticeable, rendering the affected individuals vulnerable to discrimination. This systematic review of the published literature aims to understand the socio-economic and emotional effects of SNTDs and how these are influenced by individuals’ gender. Our review includes 27 studies. Men and women experience physical suffering which reduces their ability to work, socialise, and carry out usual daily activities. Some feel well supported by partners, friends, and family, at least initially, while most are avoided, abandoned, and even suffer from verbal and physical violence, particularly women. The reported impacts of SNTDs on individuals’ emotional wellbeing include low mood, anxiety, low self-esteem, and suicidal thoughts. Early treatment, disease knowledge, social support networks, and disease acceptance were mentioned as protective factors. We conclude that SNTDs increase individuals’ psychosocial suffering, with women being particularly at risk. Ensuring treatment access and socio-economic support for men and women with SNTDs and developing culturally appropriate educational interventions designed to foster more positive attitudes towards people with SNTDs will help prevent and reduce these harms.

## Introduction

Neglected tropical diseases (NTDs) are a diverse group of twenty illnesses estimated to affect over one billion people across 149 countries [1,2]. Their burden is greatest in low-and-middle-income countries, with >70% of cases believed to occur in Africa, Asia, Latin America and the Caribbean [3]. NTDs confer devastating physical, financial and psychosocial effects. Notably, they are implicated in over 534,000 deaths and the loss of 57 million disability-adjusted life years annually [4]. Despite improvements in healthcare and sanitation resulting in reduced NTD-related mortality and disability globally, the incidence of NTDs continues to increase [3]. Impoverished communities, women and children are disproportionately affected, entrapping vulnerable individuals in a cycle of poverty perpetuated by poor health, economic and social exclusion [5,6].

Skin NTDs (SNTDs) are a subtype of at least ten NTDs characterised by their cutaneous manifestations. They include buruli ulcer, cutaneous leishmaniasis (CL), lymphatic filariasis (lymphoedema and hydrocele) (LF), podoconiosis, onchocerciasis, tungiasis, schistosomiasis, leprosy, mycetoma, scabies, fungal infections and yaws [6]. Many of these cannot be prevented by mass drug administration, and instead require individual-based diagnosis and treatment, which may be long term and often resource demanding [7]. Although rarely fatal, SNTDs have the potential to cause chronic ill-health and long-term disability, resulting in a significant number of lost disability-adjusted life years [6]. Globally, they are estimated to be the 18th leading cause of disability-adjusted life years and the fourth leading cause of disease [7]. In 2022, the World Health Organisation (WHO) published the skin NTDs framework to promote integration across different skin diseases. It was agreed that, without abandoning disease-specific focus, the distinctive and readily identifiable nature of SNTDs presents a unique opportunity for identifying synergies for integrated diagnosis, surveillance and management able to achieve greater health impact [6–8].

The framework recognises that the physical effects of SNTDs are well reported in the literature, but less attention has been paid to understanding their social and psychological implications. There are no programmes in place to support the psychosocial impacts of SNTDs and improve the quality of life of people affected with SNTDs. Yet, the visible nature of SNTDs is known to render affected individuals prone to stigmatisation, discrimination, and mental ill-health [7–8]. Despite the concept of ‘skin NTDs’ being now strategised for integrated control and management, the collective impact of SNTDs on psychosocial wellbeing has not yet been fully explored nor prioritised [6]. This represents a missed opportunity to elucidate the challenges faced by affected individuals, who may benefit from evidence-based strategic action. The improved cost-effectiveness of integrated measures is particularly important given the resource scarcity in regions with the greatest SNTD burden [9]. This paper responds to this need, and presents the findings of a systematic review conducted to bring together global empirical evidence on the psychosocial impacts of SNTDs in order to provide guidance for appropriate action.

‘Psychosocial impact’ is understood here as the effect caused by SNTDs on individuals’ social, financial, and psychological wellbeing [10], such as social stigma, economic, and emotional burdens. Mental health conditions go often undiagnosed in areas endemic for SNTDs and therefore our focus is on self-reported impacts as perceived by the affected men and women. We recognise that individuals’ mental wellbeing is heavily influenced by their immediate social and economic environment [11]. In the case of NTDs, it often manifests as emotional distress, which in turn is known to negatively influence health-seeking behaviours and treatment adherence [12]. Like other disabling diseases, SNTDs are also known to trigger various forms of stigma, all of which are associated with social exclusion, deterioration in quality of life, mental ill-health, and worse disease outcomes [13,14]. Addressing the multifaceted psychosocial burden of SNTDs is paramount to improve healthcare seeking, treatment rates, and disease control and prevention [10]–thus, to promote people’s quality of life and wellbeing.

Gender is an important concept at the interface between biological and social determinants of SNTDs. The term ‘gender’ encompasses the socially constructed roles, attributes and behaviours bestowed upon individuals on the basis of their sex [15]. Gendered differences in societal role, disease vulnerability, and care-seeking behaviour are known to influence individuals’ perceptions, experiences and treatment behaviours for NTDs and SNTDs [16,17]. For instance, the intersection of poverty and gender is known to result in health inequity for women with SNTDs due to political, socioeconomic, and cultural discrimination [18,19]. Minimal research has however looked into the influence of gender in NTD programmes and interventions [20] and even less research has been done on gendered experiences of SNTDs. While Hotez [21] noted that it is widely accepted that women’s reproductive capacity renders them particularly vulnerable to NTDs, McDonald [22] reported that pregnancy can alter NTD outcomes, causing rapid disease progression. Tsegay *et al*. [23] in turn illustrated how SNTDs are associated with high alcohol consumption among Ethiopian males, which in turn triggers intra-partner violence (IPV). What remains from the limited number of studies on gendered experiences of SNTDs is a lack of specific knowledge of how to specifically protect men or women and how to best integrate their needs and everyday life realities into people-centred public health programmes and interventions [10]. In response to this need for a gendered approach to individuals’ everyday experiences of SNTDs, this study gathers existing empirical evidence on the psychosocial consequences of SNTDs, with consideration given to the influence of gender. This will provide evidence of the lived experiences of affected individuals, thereby offering an insight into priority areas where there are opportunities for interventions.

## Methods

### Literature search

The study protocol is registered with PROSPERO (CRD42022336676) and can be found online [24]. We conducted a systematic peer-reviewed literature search using the following four electronic databases: Global Health, Embase, Web of Science, and Medline. Additional articles were identified through Google Scholar and reference tracking.

The search was restricted to full-text articles published in English between 01/01/2005 and 24/02/2024. The justification for the cut-off year relates to the formal branding of the term ‘NTD’ as a strategy to consolidate the individual disease control programmes and set the pace for global NTD initiatives [25]. Because we were interested in the lived experience narratives of individuals with SNTDs, studies were required to be qualitative in nature or have a qualitative component.

The review was conducted in accordance with ENTREQ reporting guidelines for qualitative research [26]. Eligible studies focus on one or more SNTDs. Articles pertaining to NTDs without dermatological involvement and dermatological conditions not defined as NTDs were excluded. Studies were required to include information about the social and/or psychological effects of SNTDs. Studies exclusively containing accounts from healthcare professionals, community members or family members who had not themselves had a SNTD were excluded. Participants of any age, gender and geographical region with a current or past diagnosis of a SNTD were included. Review papers and articles that are purely quantitative in nature were excluded.

The search terms included synonyms of the main search domain, SNTDs, as well as synonyms of each of the above-mentioned SNTDs recognised by the World Health Organisation. These were combined with gender-related terms and synonyms of the outcome of interest: psychosocial effects. See Box 1 below for the search syntax used in Embase:

**Box 1. Search terms as used in the Embase database**

Gender OR sex OR male* OR female* OR man OR woman OR men OR women OR girl* OR boy* AND skin AND ’neglected tropical disease*’ OR ’neglected tropical infection*’ OR NTD* OR ’buruli ulcer’ OR ’mycobacterium ulcerans’ OR leishmaniasis OR ’kala azar’ OR PKDL OR ’lymphatic filariasis’ OR elephantiasis OR podoconiosis OR ’endemic non-filarial elephantiasis’ OR ’lymphoedema and hydrocele’ OR ’wuchereria bancrofti’ OR brugia OR onchocerciasis OR ’river blindness’ OR leprosy OR ’Hansen’s disease’ OR ’mycobacterium leprae’ OR mycetoma OR ’madura foot’ OR scabies OR yaws OR framboesia OR bejel OR pinta OR ‘endemic treponematoses’ OR treponem* AND psychological OR emotional OR mental OR social OR psychosocial

* Truncation symbol, adjusted to style of bibliography.

### Screening and study selection

**Fig 1:**
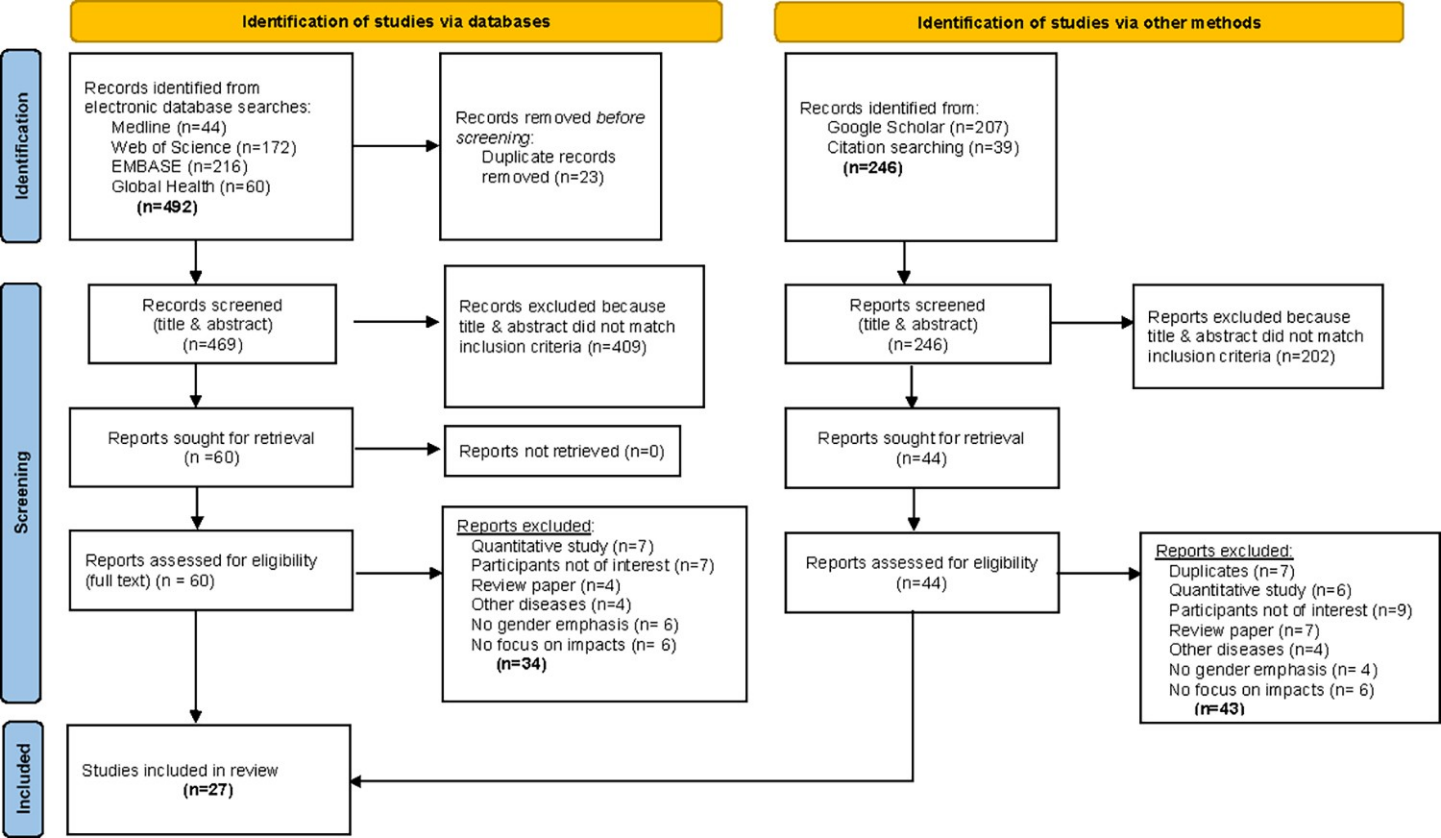
PRISMA flow diagram illustrating study identification, screening and inclusion/exclusion processes.

All articles identified through the initial search of databases and reference lists underwent a two-stage screening process by two authors (DA & MT). In the first stage, the titles and abstracts of each study were reviewed and assessed against eligibility criteria. Articles subsequently underwent full-text review. Endnote reference manager was used to facilitate the removal of duplicates and identification of eligible articles. Fig 1 summarises our identification, screening and selection process.

### Critical appraisal

The Critical Appraisal Skills Programme (CASP) quality assessment tool was applied to assess the rigour, reliability and value of all included studies [27]. Accordingly, and for the purposes of quality checking, the following information was extracted from each article: study aims, methodology, study design, recruitment strategy, data collection method(s), relationship between researcher and participants, ethical considerations and permission(s), data analysis method(s), statement of findings. DA and MT independently reviewed the quality of each included paper, and agreed on each criterion with consensus.

No validated scoring system has yet been produced for the CASP tool. In order to determine whether study authors met the specified CASP criteria studies were therefore individually scored in accordance with a system which was successfully applied to a previous qualitative systematic review [28], whereby each CASP criterion is allocated a score between zero and two (criterion completely met = 2; criterion partially met = 1; criterion not met or not mentioned = 0). Article Quality was then determined for each article based on recommendations by Abdul Rahman *et al*. [29] and Nuwagi *et al*. [30]: Total score: 20; 17–20: High Quality (>85% score); 14–16: Moderate Quality (84–70% score); ≤ 13: Low Quality (≤69%). Despite little academic consensus exists regarding how critical appraisal should be used to inform qualitative evidence synthesis, or indeed whether it is appropriate to appraise the quality of qualitative research, we agreed to exclude from the review all low-quality studies (those having ≤69% quality score). No articles were excluded on the basis of critical appraisal (see Quality checking in S1 Table).

### Data extraction and analysis

We followed a qualitative and discursive thematic narrative analysis where rather than identifying quantitative repetitions of codes, or words, we aimed to analyse the described values, narratives and perceptions of the informants. This type of exploratory thematic narrative analysis is common within cultural, sociological, anthropological, health and psychological studies that aim to understand how research participants experience a particular health or social issue [31]. To facilitate thematic synthesis, coding was carried out using NVivo software. From each study we extracted the following data: author/s, publication year, SNTD(s), geographical area, study population, number, age, and gender of participants. Additionally, all text under the heading ‘results’ were extracted and entered into NVivo software [32]. Data underwent line-by-line coding to identify key terms. Data from subsequent studies were coded into pre-existing categories, with new codes created when deemed appropriate. An inductive approach was used to generate themes which best reflect participants’ reported experiences [33]. The descriptive themes were analysed and refined, leading to the emergence of analytic themes. Authors of papers were contacted to request for missing or additional data for clarification.

## Results

### Search results

As depicted by the PRISMA flow diagram (Fig 1 above), the systematic search identified 492 studies, with an additional 246 studies retrieved through a search of Google Scholar and reference lists. 23 duplicate studies were removed, with 409 further studies excluded after title and abstract screening. The full text of the remaining 60 articles was assessed for eligibility, resulting in the exclusion of 34 studies. The remaining 26 articles are included in this review, and one additional study was located via bibliography tracking, bringing the total of included studies to 27.

### Study characteristics

The included studies were published between 2009 and 2024 and pertained to eight SNTDs: LF, buruli ulcer, CL, leprosy, onchocerciasis, tungiasis, schistosomiasis, and podoconiosis. Most are qualitative in nature, with a range of study designs used including cross sectional, mixed-methods, qualitative with ethnographic components, and phenomenological studies.

**Fig 2:**
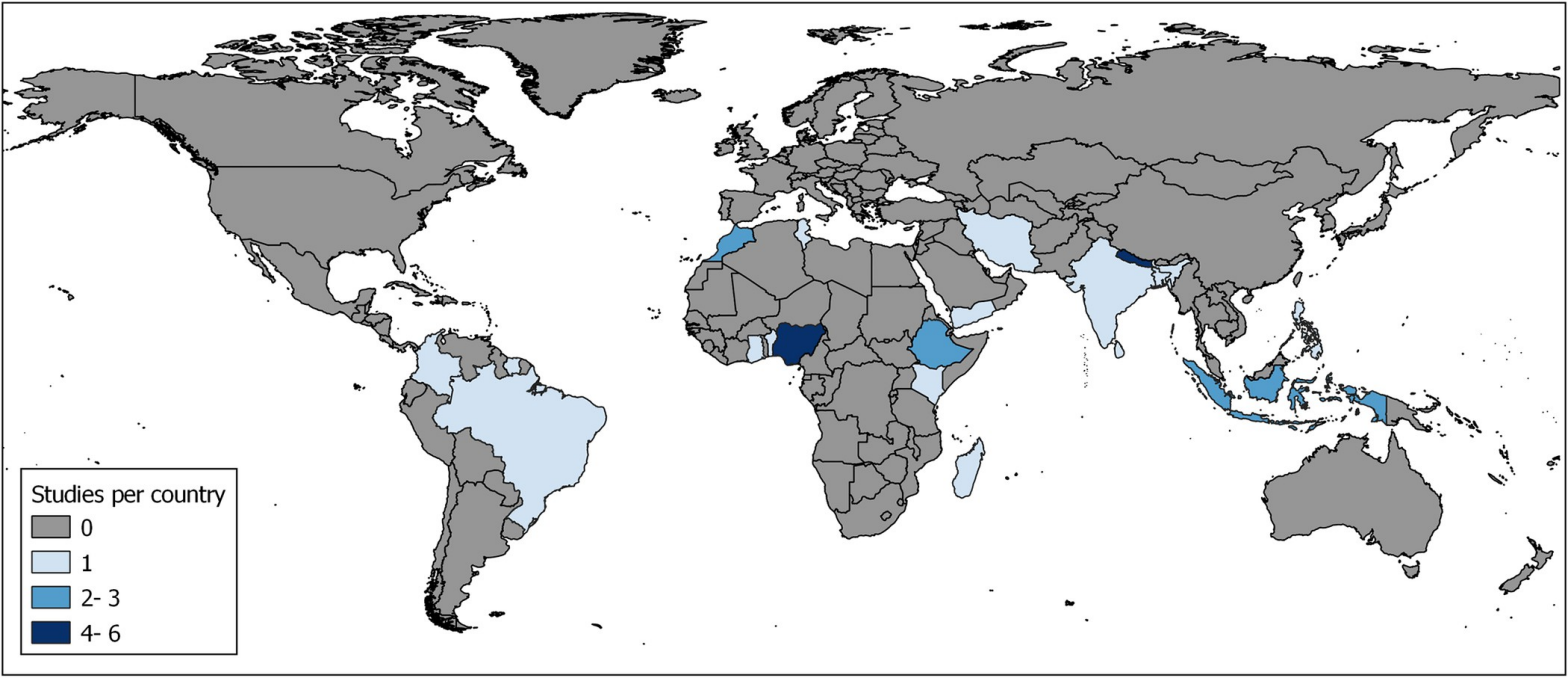
Countries of origin of included studies. The map was produced using QGIS mapping software (https://qgis.org). The shapefile for the basemap is from the GADM database of Global Administrative Areas, version 4.1. Shapefiles are available for download from GADM at: https://gadm.org/.

The included studies report findings from a total of 19 countries, with two articles reporting findings from various countries. Taking the World Health Organization (WHO) regions as reference point, of the 27 articles, eleven (40.7%) report findings from the African region, three (11.1%) from the Americas, five (18.5%) from the Eastern Mediterranean region, ten from South-East Asia (37%), and one from the Western Pacific region (3.7%). The geographical distribution of the included studies is illustrated in Fig 2 below.

### Quality of the included studies

Identified studies are of high and moderate quality, scoring between 14 and 20 based on the CASP checklist (see S1 Table). Twenty-five (92.6%) studies were deemed to be high quality (>85% CASP score), and two (7.4%) moderate (84–70% CASP score). None of the articles were deemed to be of low quality (≤69% CASP score). Each paper clearly stated its aims and employed an appropriate qualitative study design. Additionally, all studies collected data in a manner which addressed the research issue and produced a clear statement of findings. Twenty-five (92.6%) studies appropriately recruited participants and explicitly discussed relevant ethical considerations. All of the included studies were deemed to have rigorously analysed their data, thereby producing valuable results. Only nine (33.3%) studies fully considered reflexivity, in terms of the participant-researcher relationship.

### Review findings

In accordance with the inductive data analysis, we have organised the results in four overarching themes: a) SNTD symptoms triggering negative psychosocial impacts, b) Psychosocial impacts on marital relationships, c) Psychosocial impacts on wider inter-personal relationships, d) Impacts of SNTDs on individuals’ emotional and mental wellbeing. Across these themes, findings also illustrate the ways in which overlapping local scarcities and expectations on what men and women should be and do intersect with bodily SNTD effects to influence gender distinctions in the psychosocial impacts of SNTDs. A summary description of the included studies is featured in Table 1 below. A more comprehensive description of the data disaggregated by gender, SNTD, and endemic country can be found in S2 Table.

**Table 1:** Characteristics and quality of included studies.

Study	Country	Skin NTD	Qualitative methods of data collection	Number of participants	Gender	Age	Article Quality
**Abdulmalik *et al*. 2018 [34]**	Nigeria	Lymphatic Filariasis	Interviews & Focus Groups Discussions	69	32 men, 37 women	20–80	High
**Adekeye *et al*. 2023 [35]**	Nigeria	Buruli Ulcer, Lymphatic Filariasis, Leprosy	Photovoice	14	8 men, 6 women	18-≥49	High
**Agbo *et al*. 2019 [36]**	Benin	Buruli Ulcer	Interviews, illness narratives, case studies	45	45 women	Not specified	High
**Al-Kamel 2017 [37]**	Yemen	Cutaneous Leishmaniasis	Interviews	11	1 man, 10 women	12–60	Moderate
**Arjyal *et al*. 2023 [38]**	Nepal	Lymphatic Filariasis	Interviews & Focus Groups Discussions	39	19 men, 20 women	18–70	High
**Azubuike *et al*. 2023 [39]**	Nigeria	Buruli Ulcer	Interviews & Focus Groups Discussions	14	8 men, 6 women	31–60	High
**Bennis *et al*. 2017 [40]**	Morocco	Cutaneous Leishmaniasis	Focus Groups Discussions	247	120 men, 127 women	19–82	High
**Bennis *et al*. 2017 [41]**	Morocco	Cutaneous Leishmaniasis	Open questionnaire	448	258 men, 190 women	11–20	High
**Boukthir *et al*. 2020 [42]**	Tunisia	Cutaneous Leishmaniasis	Interviews	10	4 men, 6 women	27–65	High
**Bow-Bertrand *et al*. 2019 [43]**	Bangladesh	Leprosy	Questionnaire, Interviews	30	17 men, 13 women	18–71	High
**Dako-Gyeke *et al*. 2017 [44]**	Ghana	Leprosy	Interviews	26	16 men, 10 women	40–90	High
**Eneanya *et al*. 2019 [45]**	Nigeria	Lymphatic Filariasis	Open Questionnaire, Interviews	52	36 men, 16 women	21-≥60	High
**Khatami *et al*. 2018 [46]**	Iran	Cutaneous Leishmaniasis	Interviews	12	6 men, 6 women	23–63	High
**Milallos & Basubas 2019 [47]**	Philippines	Leprosy	Interviews, Observation	5	2 men, 3 women	37–69	High
**Noordende *et al*. 2016 [48]**	Nepal	Leprosy	Interviews	10	10 women	22–50	High
**Noordende *et al*. 2020 [49]**	Ethiopia	Leprosy, podoconiosis, Lymphatic Filariasis	Interviews & Focus Groups Discussions	57	Not specified	17–73	High
**Nuwangi *et al*. 2024 [10]**	Sri Lanka	Cutaneous leishmaniasis	Participant Observation, Interviews, & Auto-ethnographic diary	30	14 men, 16 women	18–75	High
**Otache *et al*. 2023 [50]**	Nigeria	Onchocerciasis	Focus Groups Discussions	32	16 men, 16 women	30–66	High
**Putri *et al*. 2023 [51]**	Indonesia & India	Leprosy	Interviews	66	49 men, 17 women	16-≥49	High
**Ramdas *et al*. 2016 [52]**	Suriname	Cutaneous Leishmaniasis	Interviews, Observation, open-ended questionnaire	224	192 men, 32 women	19-≥ 50	High
**Schuster *et al*. 2023 [53]**	Madagascar	(genital) Schistosomiasis	Interviews & Focus Groups Discussions	76	76 women	15–35	High
**Stephen, K. 2023 [54]**	Kenya	Tungiasis	Interviews & Focus Groups Discussions	46	22 men, 24 women	18–60	High
**Susanto *et al*. 2023 [55]**	Indonesia	Leprosy	Interviews	9	9 women	21–42	High
**Tsegay *et al*. 2018 [23]**	Ethiopia	Podoconiosis	Interviews	15	15 women	21–75	High
**van Netten *et al*. 2021 [56]**	Nepal	Leprosy	Interviews	14	4 men, 10 women	35–70	High
**van Wijk *et al*. 2021 [57]**	Colombia	Leprosy, Cutaneous Leishmaniasis	Interviews & Focus Groups Discussions	47	28 men, 19 women	28–69	High
**Varkevisser *et al*. 2009 [58]**	Indonesia, Nigeria, Nepal, Brazil	Leprosy	Interviews & Focus Groups Discussions	154	71 men, 83 women	Not specified	Moderate
			**Total**	1802	923 men, 822 women	11–90	

### SNTD symptoms triggering negative psychosocial impacts

**Fig 3:**
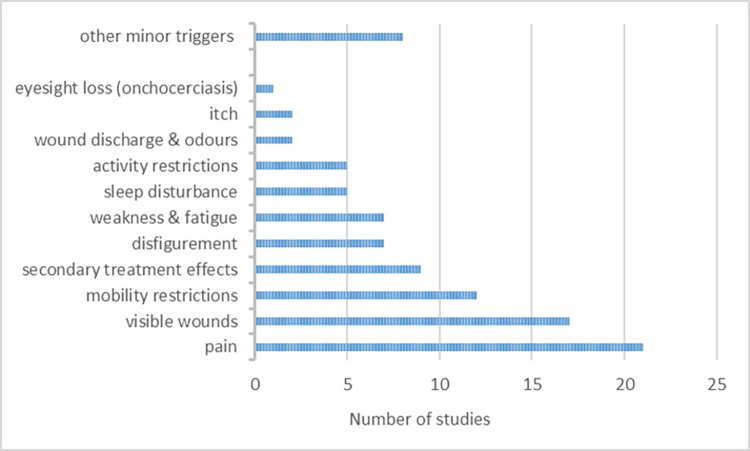
Physical SNTD symptoms triggering negative psychosocial impacts as reported by the affected persons.

Men and women in 25 (93%) studies reported experiencing psychosocial difficulties directly related to the physical symptoms of their SNTDs. Across, contexts, genders, and SNTDs, the most frequently mentioned physical symptom was pain, reported in 21 (78%) of the included studies (Fig 3 and S2 Table). Other SNTD symptoms that participants indicated triggered negative psychosocial impacts included: visible wounds and lesions (63% of studies), mobility restrictions (44%), disfigurement (26%), fatigue (26%), sleep disturbances (19%), and wound odours (7%). Minor symptoms also mentioned to trigger negative psychosocial impacts across genders and SNTDs included numbness, swelling, fever, nauseas and vomits, stiffness, and general weakness [39,50,55,56]. Eyesight loss was also reported to cause important negative emotional and socio-economic impacts for Nigerian men and women with onchocerciasis [50].

Men and women across contexts and SNTDs commonly reported that, combined, the physical symptoms of their SNTD significantly impaired their ability to socialise and carry out their normal everyday activities (reported in 93% of the studies). For instance, 63% men and women with leprosy, podoconiosis and LF living in Ethiopia reported activity limitations, 37% of them describing these as severe [46]. In this study, 69% of respondents reported that they were unable to work, and 13% indicated that whilst they continued to work, they were unable to do so in the same capacity [49]. Seventeen (63%) of our included studies explicitly mentioned job loss or dismissal as a result of having a SNTD. This seemed to affect men slightly more than women, perhaps because women are often responsible for childcare and managing the household [36,53]. Equally affecting men and women, 13 (48%) studies explicitly associated having a SNTD with household impoverishment, with men and women often depending on the goodwill of others for their survival [36].

Individuals’ decreased ability to work was not only influenced by disease-specific impairments, but also by the visibility of the skin lesions and associated fear of infection and stigma. Several participants across SNTDs and geographical regions felt that their lesions were unsightly, particularly women [10,40,41,55]. For instance, a Moroccan woman with CL explained that scars located centrally on the face were particularly troublesome, as they could not be concealed with a veil [40]. A Ghanaian woman also explained the difficulties posed by her leprosy-related impairments as follows:

“I don’t think anyone will employ a person with leprosy (…) so for me, I won’t even look for a job because of my visible impairments. If people who are healthy don’t have jobs, how much more those of us who are not healthy? We made efforts to look for jobs, but were advised not to waste our time. Let’s assume you were offered a job and you don’t have fingers and toes, how can you work?” [44]

(Ghanaian woman with leprosy)

Fearing rejection and discrimination, across contexts and SNTDs men and women with visible lesions often deterred from socialising and from looking for jobs. A man with leprosy highlighted the detrimental impacts that inability to work had on his mental wellbeing, describing this as his primary disease-related concern:

“I’m not worried about my disease. I am worried about my foot. Without it, I could not work like other normal people. I feel jealous when I see other normal people” [56]

(Nepali man with leprosy)

Nine (33%) studies also suggested that treatment of SNTDs was often associated with significant physical, emotional and financial hardships, especially in the case of CL. For instance, men and women living in Tunisia, Iran and Colombia reported that the side effects of CL treatment were extremely severe, and often described the treatment as being more unpleasant than the disease itself [42,46,57]:

“After 2 injections, my sores got worse…I went to the bathroom crawling. The injections that I received in the lesions were painful, they didn’t work, my leg became more inflated.” [42]

(Tunisian man with CL)

A Colombian man even described CL treatment as “psychologically most affecting” [57].

The financial implications of accessing treatment were also cited as a cause for concern for men and women in nine (33%) studies. Various Madagascan women with schistosomiasis became indebted [53]. Likewise, a Bangladeshi man with leprosy reportedly spent around half a million TK (approximately £4400 GBP) on treatment, forcing him to sell his land and home [43]. A Nigerian man also stated the following:

“I’ve depleted my income and savings for myself and my children in trying to cure my condition.” [45]

(Nigerian man with LF)

This highlights the damaging effects of seeking treatment on family economies, with wide-ranging secondary effects affecting the entire household.

### Impacts on marital relationships

Nineteen (70%) studies reported on the effects of SNTDs on informants’ relationships with their partners (S1 Table). These ranged from increased support and closeness to avoidance, infidelity, abandonment, and intra-partner violence.

#### Avoidance, infidelity and abandonment

Seventeen (63%) articles indicated that having a SNTD was associated with a decline in relationship quality. Men and women in fifteen (56%) studies reported falling victims of abandonment or infidelity, with women being more vulnerable than men. Avoidance, described in thirteen (48%) studies, normally preceded infidelity and abandonment [23,34,41,45,48,57]. The included studies primarily provided accounts of women being avoided by their male partners (mentioned in 12 studies), with six studies describing avoidant behaviour by the female partners. Commonly, women felt that their male partners had grown distant and unsatisfied with the relationship, opting to distance themselves from them and, in some cases, pursue sexual relationships with others [23,45,48,57].

“When I was diagnosed, I felt that my husband’s behaviour had changed, he didn’t share anything with me and he pretended to be busy with work. But actually he was trying to be far away from me” [48]

(Nepali woman with leprosy)

Three papers attributed avoidance to the male partners’ fear of infection [34,48,57]. Across contexts, women also expressed concerns regarding potential disease transmission to partners and close family members [40,48,52,57]. An extreme instance of this is exemplified by a Colombian woman who left her husband without telling him about her leprosy due to her perceived need to self-isolate to protect loved ones [57].

Fear of disease transmission was also reported to negatively impact intra-marital sexual relationships [23,34,41,45,57]. Several women reportedly abstained from sex entirely since their diagnosis [23,45,48], and 47% Ethiopian women with podoconiosis reported that their husbands avoided sexual intercourse with them and instead sought sexual contact elsewhere [23]. Disease transmission misunderstandings often affected marital sexual relationships:

“My husband is afraid that it transmits through respiration, so he doesn’t want to tongue kiss for seven months” [48]

(Nepali woman with leprosy)

Two Nigerian males with LF also reported no longer sexually engaging with their wives due to shame and general SNTD-related relationship decline [45]. In turn, Varkevisser *et al*. [58] highlighted that whilst Nigerian and Indonesian men often discontinued sexual intercourse with their sick partners for the first few months after diagnosis until leprosy symptoms started to subside, women enjoyed less freedom to do so.

Avoidance and infidelity commonly preceded outright rejection and abandonment [23,36,37]. A study assessing the impact of leprosy, podoconiosis and LF on family quality of life in Ethiopia found that 20% participants of both genders divorced due to the SNTD or were continuously asked for divorce by their partner [49]. In turn, Varkevisser *et al*. [58] calculated that about 25% of the married women with leprosy in Indonesia and Nigeria reported abandonment or divorce compared with 10% of men. Commonly cited reasons for abandonment and divorce included pressure from a partner’s family [34,40], the affected individual’s inability to fulfil their designated role [23,36,45], and the husband’s desire to marry a “healthier woman” [23, 57]. In turn, the implications of abandonment and infidelity for women were wide-ranging, from emotional distress to financial hardship, indebtment, and a risk of exposure to sexually transmitted infections [23,36,49,57].

#### Intimate partner violence and abuse

None of the male participants in the reported studies experienced violent or abusive behaviour at the hands of their female partners, but IPV and abuse was reported by female participants in four (15%) studies [23,36,48,56]. Abuse took various forms, ranging from insults and controlling behaviour to physical and sexual violence, including restricting access to financial resources, social networks, employment, and healthcare. In northern Ethiopia, women with podoconiosis commonly reported being pressured into sexual acts, with threats of abandonment, violence and cessation of support if they failed to comply [23]. Similar abuses were also reported by women living in Nepal:

“When I don’t want to have sexual intercourse, my husband forces me. He scolds me ‘I earn money, bring food for you all but you don’t want [to have sex], then get out of the house!’ Sometimes he raised a hand on me…so I have to be near and close and have sex with him” [48]

(Nepali woman with leprosy)

A study exploring IPV against women with podoconiosis in northern Ethiopia found that IPV predated the onset of podoconiosis in 87% of cases [23]. Although not the primary determinant of abuse, podoconiosis was associated with an increase in the frequency and severity of IPV:

“His violence increased as the disease aggravated. The problem is the disease because it hinders my ability to work hard” [23]

(Ethiopian woman with podoconiosis)

This study concluded that the increased violence arose due to men’s frustration and dissatisfaction with being married to “unhealthy” women who failed to fulfil their expected roles, which was confirmed by an informant who stated:

“… arriving home he says ‘while I am working hard, you only sit at home, and I shouldn’t feed such a weak woman’. He used to attack me with whatever he found around him just like a knife or even an axe. If he got drunk, he would use anything” [23]

(Ethiopian woman with podoconiosis)

Across contexts and SNTDs, most of the women who had experienced domestic violence had not received formal schooling and were unemployed and financially dependent on their husbands [47,56]. This dependence rendered them vulnerable to violence and limited their ability to leave the relationship. As illustrated by an Ethiopian woman with podoconiosis, the partner’s financial support was often contingent on their expectations being met:

“…he was always complaining at everything, saying ‘why didn’t you do this and this?’ and I was telling him ‘you know that I can’t do all the activities in the house and outside the house (in the farmland).’ He used to beat me and say ‘if you can’t, go away from the house!” [23]

(Ethiopian woman with podoconiosis)

In turn, the expectation to fulfil childcare, household and work duties under the threat of violence prevented some women from resting and elevating their legs, resulting in worsened disease outcomes in podoconiosis [23]. This was exacerbated by the fact that some men obstructed their partner’s access to healthcare, and/or refused to provide them with resources required for self-care [36]:

“When I begged him to take me to the health centre, he always made excuses. Then the disease got worse as a result of the delay” [23]

(Ethiopian woman with podoconiosis)

#### Difficulty finding partner

Across geographical locations, males and females with SNTDs reported struggling to find a partner, resulting in delayed marriage in contravention of local social norms [34,37,40,41,44,45]. This issue was reported in eleven (41%) studies, 82% of which reported on women’s experiences and 54% on men’s. A man with LF recognised a correlation between disease severity and difficulty finding a marital partner explaining:

“When the condition isn’t severe, then a person can get married without much difficulty. But when the sickness is very severe it can be a deformity and no girl will want to marry you” [45]

(Nigerian man with LF)

Whilst both male and female participants reported difficulty in finding a partner because of their disease, women seemed to be more susceptible to encountering this problem than men, partly because of the common depiction of males as breadwinners [34,37,40,41,45]. In their study investigating CL in Morocco, Bennis *et al*. [40] describe the emotional distress most commonly reported amongst women whose marital prospects had been limited by their SNTD. The following quote illustrates the gender disparities of this social risk as perceived by a Moroccan woman:

“She’ll be afraid about her future especially for the wedding. Meanwhile, in our society, an affected boy remains a man. There is no harm if he has scars” [41]

(Moroccan woman with CL)

#### Positive responses from partners

Men and women in ten (37%) studies reported that being diagnosed a SNTD had not caused any decline in their marital relationships:

“My wife married me not minding my condition. She has lived and supported me ever since we got married.” [45]

(Nigerian man with LF)

Whilst data indicates that women tended to be more supportive of their partners’ needs, some women in nine (33%) studies also reported being well-supported by their male partners:

“My husband watched over me; he came [with me] to the clinic every day” [57]

(Colombian woman with CL)

Varkevisser *et al*. [58] also described how young Nepali husbands decided to keep the disease and treatment of their wives (all without visible deformities) hidden from their parents in order to save their marriage. Agbo *et al*. [36] however suggested that, at least in the case of Beninese women with buruly ulcer, husbands’ support was only temporary: when requests for time or resources exceeded the husbands’ capacity to provide, social bonds and support were weakened.

### Impacts on wider inter-personal relationships

#### Avoidance, violence and rejection from relatives

Across study sites, men and women with SNTDs primarily relied on their partners, children or relatives for psychological, financial, and practical assistance. As explained above, avoidance, abandonment and rejection by partners was common. Male and female participants in twenty-one (78%) studies also reported being avoided or abandoned by family members. Of these studies, 92% described abandonment of women and 77% the abandonment of men. It is unclear how frequently this happens, but a study focused on leprosy in Indonesia and Nigeria reported that 25% women felt that close relatives avoided them, in contrast to only 4% of the men who experienced such behaviour [58]. A woman with buruli ulcer expressed her disappointment at being abandoned by her sister as follows:

“When I came here to seek treatment, I gave my big sister all my savings and belongings to hold for me while in the hospital. However, she abandoned me in the most difficult moments of my life (…) nasty, no?” [36]

(Beninese woman with buruli ulcer)

Likewise, a Bangladeshi woman with leprosy remarked that she “sometimes felt some degradation of dignity” from her family [43]. This was echoed by men and women in various countries in several other studies, who reported often feeling ostracised, stigmatised and shunned by family members [36,42–44,49,57]:

“I had to move to this Leprosarium because my parents and siblings felt the disease made me a disgrace to the family” [44]

(Ghanaian man with leprosy)

Another Ghanaian man highlighted the profound impact that lack of familial support had on his mental wellbeing, stating:

“before I moved to the leprosarium, sometimes I felt like killing myself because of this sickness… My family didn’t care about me, even getting a place to live and food to eat was a problem” [44]

(Ghanaian man with leprosy)

A Kenyan man even admitted that he could easily die in his home because he could not feed himself, no one visited him and he could not walk.

‘I feel so lonely’ [54]

(Kenyan man with tungiasis)

In some cases, lack of disease-related knowledge was a contributory factor to discrimination, as highlighted by a Colombian woman whose sister initially rejected her, until finding out that leprosy is a curable disease [57]. Other mentioned reasons for family avoidance included fear of infection, perceived association between SNTDs and sinfulness, economic uncertainty, caregivers’ strain, and anticipation of social rejection. Rejection was often preceded by conflict, verbal, and physical violence [23]. Of the nine (33%) studies mentioning violence from family members, eight (89%) specified violence towards women and 4 (44%) towards men. Relatives’ violence towards women with SNTDs seemed to take place across WHO regions, whilst violence against men with SNTDs was more frequently reported in the African WHO region (75% of articles, see S2 Table).

#### Positive family responses

Male and female respondents in nine (33%) studies reported feeling supported by family members other than their partners. Positive family responses included moral or emotional support, providing money or other resources, taking over household duties such as cooking and washing clothes, and helping with self-care. It is unclear how frequently this occurs, but in the Ethiopian study exploring the impact of SNTDs on family quality of life, 54% of participants felt well-supported by family members, with 21% expressing that they received no or limited familial support [49]. The encouragement and social support provided by family members was often mentioned as being instrumental to recovery:

“It was my family members that have been washing and dressing my wounds each morning (…) our children were very happy for me and were even singing out of joy when I eventually began walking with the aid of a walking stick after eight months of being bed-ridden” [34]

(Nigerian woman with LF)

Likewise, a Nepali man with leprosy praised his son for encouraging him to seek treatment, which proved to be greatly beneficial to his physical and mental health [56]. On rare occasions, contracting a SNTD even conferred positive effects to some respondents, having brought them closer to their family [57].

Despite the benefits of familial support, respondents in ten studies (37%) experienced distress due to the material and anticipated repercussions of their SNTD on their family members see for instance [36,37,49,56,57]. The most commonly mentioned consequences of SNTDs on supportive family members were a decrease in earnings and impoverishment, caregivers’ strain (particularly for girls and women), discrimination (e.g. job dismissal, social ostracism), disease onset, and a decrease in school attendance and of marital prospects for descendants [37,46,48,49]. These were due to a combination of income insecurity, the assumed associations between SNTDs and sinfulness, and the belief that the disease ‘runs in the family’ or is somehow ‘deserved’ [23,57]. The complex and potentially wide-ranging negative impacts of SNTDs on supportive family members were for instance illustrated by Agbo *et al*. [36], who explained how a five-year-old girl became the main carer of a Beninese mother with buruli ulcer, providing her mother with extensive support by going to the market, cooking, washing clothes and managing her mother’s medication. Although greatly appreciative of the support, the mother expressed concern regarding her daughter’s future, which she felt she had jeopardised:

“If I have to remain in the hospital longer, what will happen to her? While she is very intelligent, it will be hard for her to succeed in school” [36]

(Beninese woman with buruli ulcer)

Among the included studies, three women reportedly abandoned their supportive families to protect them from the consequences of family care [57].

#### Societal avoidance, discrimination, and violence

In 18 (67%) studies, men and women across contexts and SNTDS commonly reported being avoided by community members, or altogether rejected by society. Some spoke of being barred from weddings, gatherings and neighbours’ homes [45,49]. Many were also subjected to gossip, unwanted attention, and stares [58], with men and women often feeling so uncomfortable that they opted to socially withdraw themselves [34,41–43,45,46,57]. In various instances community members were actively hostile towards those living with SNTDs, a SNTD reaction reported in seven (26%) of the studies [34,40,44,47,49,52,56]:

“They become afraid of us, and others even insult us… Some of them spit out saliva when they see us [in disgust or revulsion]” [34]

(Nigerian man with LF)

Occasionally, hostility was so severe that individuals with SNTDS were shunned or evicted from communities [44,47], especially in the case of leprosy:

“When I went home, I was like a convict that people would shut their windows whenever I pass by their house… Our *barangay* [community leader] approached me and petitioned to evict me from our community. Finally, they had a petition to evict me signed by my neighbours.” [47]

(Philippine man with leprosy)

Similarly, Ghanaian men and women were reportedly forced to live in isolation in forests, because it was considered taboo for people with leprosy, including those who had been cured, to live in the same place as non-affected people [44].

According to Ariyal *et al*. [38], Nepali women with LF faced more social societal stigma and discrimination compared to males because of the perceived value of men as breadwinners in the patriarchal society. Varkevisser *et al*. [58], also stated that Nepali women with leprosy suffered most from societal discrimination than men, but highlighted that deformed men had also been chased to the outskirts of the villages, where they were also neglected until they died.

Men and women with SNTDs also encountered instances of institutional discrimination, including stigmatisation by healthcare workers [57], being asked to leave restaurants, denied accommodation and jobs, dismissed from work, and not allowed on public transport [44,52,54,57]:

“One day, I went to eat at a restaurant and when other customers saw me, they started leaving the restaurant. One of the workers at the restaurant requested me to leave else they would lose their customers. I was not served” [44]

(Ghanaian man with leprosy)

Often, societal and institutional discrimination was not limited to individuals having SNTDs; it often extended to partners and family members, especially those who were supportive [23].

### Impacts of SNTDs on individuals’ emotional and mental wellbeing

26 (96%) studies reported on the negative emotional impacts of SNTDs on men and women. The prevalence of mental ill-health associated to SNTDs is not known, but 54% Ethiopian men and women with leprosy, podoconiosis and LF reportedly experienced a mental health issue [49]. Across genders, SNTDs and contexts, self-reported negative emotional impacts ranged from worry, low mood and low self-esteem to anxiety and suicidal ideation.

#### Low mood, depression, and suicidal thoughts

The most commonly described SNTD impact on men’s and women’s mental wellbeing was low mood, reportedly affecting women in 19 (70%) of the included studies and men in 14 (52%). Across contexts and SNTDs, men and women frequently cited disfigurement as a principal factor contributing to their low mood [34,40,41,46,47,52]. Low mood was often accompanied by low self-esteem due to the aesthetic stigma associated with SNTD lesions, which many men and women described as “disgusting” or “a curse” [42,46]. For instance, males and females from Morocco and Ethiopia expressed that their physical disfigurement made them feel inferior to their unaffected counterparts and diminished their value [41,49].

Low mood among men and women was also due to the social reaction they received from others due to their SNTD, which, as explained earlier, often ranged from low-level microaggressions and unwanted attention to overt hostility [34,40,43,44,47]. In turn, van Netten *et al*. [56] also highlighted that Nepali women frequently experienced depression as a result of the difficulties arising from their economic dependence on their husbands, and concluded that in Nepal, leprosy severity and disability grade correlated to poorer psychological outcomes.

Our research also indicates that the negative emotional impact of SNTDs was exacerbated by the associated physical symptoms and complications [34,42,56,57]:

“I feel demoralized and very sad. There was a time that I was in severe pain and I prayed to God to just take my life so that I will be relieved of the pain.” [34]

(Nigerian man with LF)

The pervasive nature of SNTD lesions was also reported to be a further driver of chronic low mood and mental distress [41,47]:

“The psychological state of the affected person can worsen after receiving treatment because the problem is that scars never disappear” [41].

(Moroccan man with CL)

A Ghanaian man also described the pervasive nature of SNTD-related stigma and disability as follows:

“Many people, including my family members don’t believe I am cured because of the deformities, especially the sores on my leg” [44].

(Ghanaian man with leprosy)

This meant that discrimination and isolation often continued after the disease was cured. A Nigerian man for instance admitted that he drunk alcohol to ease off the long-term consequences of onchocerciasis [50].

Suicidal ideation was a common consequence of SNTDs across genders and geographical locations. It was reported in 11 (41%) of the studies, more frequently in the case of men (8 studies) than women (7 studies). Reasons cited included pain, loss of employment, fear of the future, burden on caregivers, violence, and social rejection [34,37,42–44,51]. Religious beliefs were occasionally cited as a deterrent from suicide:

“…if people die by ending their own life, they must go to Hell” [43].

(Bangladeshi woman with leprosy)

In contrast, several Nigerian men and women with LF spoke of praying to God to end their lives:

“There was a time that I was in severe pain and I prayed to God to just take my life so that I will be relieved of the pain” [34].

(Nigerian man with LF)

#### Feelings of excessive worry, stress, and anxiety

Seventeen (63%) studies indicated that males and females across WHO regions and SNTDs experienced feelings of anxiety, excessive worry and stress pertaining to their SNTD, 88% of which described experiences of women and 59% of men. These feelings primarily related to their inability to work and resultant financial hardships [34,43]. In some cases, this financial hardship was exacerbated by the expenses incurred through costly treatments, as exemplified by the case of a Bangladeshi man with leprosy, who after spending most of his money on treatment worried about the future survival of his business [43]. Other treatment-related concerns equally affecting men and women in Yemen, Morocco and Colombia related to the perceived lack of available treatment and concerns related to the nature of treatment itself [37,41,57]. Lack of information, incomplete information, and inconsistent messages from health professionals about the prognosis of leprosy further generated anxiety and despair among Indonesian and Indian men and women [51]. Stigmatisation (real and anticipated) was also frequently cited as participants’ biggest source of emotional distress [44,46,49,56]. Men and women across WHO regions also expressed concerns related to fear of deformity, disease complications and death, as well as fear of the negative social response associated with SNTDs [37,41,42,45,46,57].

Anticipated stigma was also a key source of anxiety, as illustrated by a Tunisian man with CL who described himself as “exhausted” by his “overwhelming apprehension of being rejected by society” [42]. Among Indonesian and Nigerian men, fear of the stigma attached to leprosy also served as an encouragement to seek treatment quickly before deformities could develop [58].

#### Embarrassment, frustration, and anger

Men and women in nine (33%) studies equally reported feeling frustration or anger at being ill and incapacitated, as well as anger for the abandonment, discrimination and stigmatisation to which they had been subjected. Men and women also commonly reported feeling embarrassed or ashamed of their condition [34,37,40,42,45,46,56,49,52]:

“my disease hurts me and makes me shameful’” [56]

(Nepalese woman with leprosy)

This was slightly more frequent in men (S1 Table), and closely linked to aesthetic stigma and self-stigmatisation, as well as to experiences of discrimination and job loss. In turn, individuals who felt ashamed of their lesions were more likely to isolate themselves and withdraw socially, conferring wider negative psychosocial and economic effects [34,37,44,45,49,56,57].

#### Low self-esteem and self-stigmatisation

Self-stigmatisation, described in nineteen (70%) studies, was often developed as a result of low self-esteem, influenced in turn by the symptoms of the disease, fear of contagion, burden on carers, and experiences of abandonment, abuses, and discrimination. As explained earlier, men and women often felt self-conscious about their lesions, which mostly women described as “ugly”, “cosmetically embarrassing” and “disgusting” [37,45,46]. This often made them feel inferior to their unaffected counterparts [44,57]. Several participants even confessed to empathising with the stigmatisation and discrimination they had experienced:

“People in the wedding made me stay outside of the tent and they did not treat me as (if) I was healthy… How can I be equal with this disease? I sometimes agree with what they did.” [23]

(Ethiopian woman with podoconiosis)

In turn, internalised stigma prompted some individuals, primarily women, to distance themselves from their community despite being treated “normally” by others [52].

#### Disease acceptance and mental wellbeing

Mental wellbeing was associated with disease knowledge, acceptance and social support. Participants in nine (33%) studies expressed that they had not experienced negative emotional effects [34,37,40,42,45,46,49,52,56]. Most were men who had not experienced stigmatisation or discrimination and felt their condition was “normal” [37,45,56,49,52], or patients who were reassured by their doctors [57]. The perception that a disease was curable and non-contagious also influenced disease acceptance and reduced the negative psychosocial impacts of SNTDs [40]:

“no one will reject you or refuse you because you have *busi yasi* [CL]… and it is really not a sore to be afraid of, because it is curable. It is also not contagious, because otherwise everybody in the village would get it, right?” [52].

(Surinamese man with CL)

Al-Kamel *et al*. [37] in turn observed that, in general, younger Yemeni generations affected by CL showed less acceptance than older adults did.

Various men admitted that having a visible SNTD scar did not confer distress:

“What’s the matter to have an additional scar? It’s normal! Even for women, it is the signature stamp of our area” [40]

(Moroccan man with CL)

Whilst generally female participants were less accepting of their SNTD and associated lesions, those at the extremes of age were less liable to stigmatisation:

“I don’t experience any social or aesthetic stigma because I’m old” [37]

(Yemeni woman with CL)

For Ghanaian and Philippine men and women with SNTDs, feeling accepted and socially integrated in their environment helped them accept the physical impacts of their disease and promoted mental wellbeing [44,47]. Participants in several studies also described the emotional benefits of receiving help from their community in the forms of encouragement, emotional support and financial assistance [34,43,45–47,49,52,56]. Various studies also emphasised the protective effect of knowledge:

“I don’t feel stigma (…) community members know about this condition and what causes it” [45]

(Nigerian man with LF)

The belief that a SNTD was the will of God also often caused some individuals to feel that there was no reason to be ashamed or afraid [34,37,47,56]:

“God gave me this, so why should I feel bad about leprosy?” [56].

(Nepali woman with leprosy)

However, various studies also described how the widely-held belief that SNTDs were a punishment which afflicted those who had sinned also caused damaging self-stigma, social discrimination and blame [23,43,44].

## Discussion

This review has sought to understand the psychosocial impacts of SNTDs as perceived by the affected men and women in order to explore potential gender differences and inform public health interventions for prevention and mitigation. Men and women across WHO regions reported disfiguring lesions and debilitating physical symptoms associated with their SNTDs or their treatment. These commonly resulted in functional impairment and isolation, as well as in reduced ability to work and to fulfil expected household chores and childcare responsibilities. Described by men and women as their primary disease-related concern, inability to work and decreased ability to conduct usual daily activities were associated with significant emotional, social, and financial ramifications. In some cases, these were reportedly severe, with suicidal ideation reported by participants of both genders as a result of loss of employment [34,43]. These findings support those of Kuper *et al*. [59], who established a link between NTD-associated disability and adverse psychological effects.

Our review has not identified gender differences in the emotional ramifications of SNTD-related unemployment. However, previous NTD research concludes that the detrimental psychological effects of unemployment are generally more pronounced in men, owing to gendered social norms and the expectation for men to adopt the traditional role of the “breadwinner” [60]. Our findings however agree with wider NTD literature in that disease-related inability to fulfil expected gender roles is amongst the most frequently reported reasons for stigmatization [61]. As we have shown, individuals decreased ability to fulfil expected gender roles influences instances of abandonment and violence from partners and relatives. This is more apparent in the case of women, who frequently reported that their partners’ behaviour towards them was contingent on their ability to meet gendered expectations [23,36,48,56]. In turn, the need to fulfil childcare, household and work duties under the threat of violence or eviction prevented some women from resting and elevating their legs, resulting in worsened disease outcomes in podoconiosis [23]. Women commonly reported experiencing physical and emotional abuse from partners and relatives, with the level of violence commonly correlating to disease severity [23], and length of symptoms [36].

Across contexts and SNTDs, the issue of violence against women is heavily compounded by the power imbalance and unequal social standing of men and women in the endemic regions [62,63]. As noted in various studies, most of the women who had experienced domestic violence had not received formal schooling and were unemployed and financially dependent on their husbands [23,47,56]. This rendered women vulnerable to abuse and its profound psychological effects, and also had important implications for their dependants. As shown, women with SNTDs also often faced emotional difficulties arising from their economic dependence on their husbands, frequently experiencing feelings of depression and low self-esteem as a result [56]. In addition, whilst none of the included studies reported instances of IPV towards men with SNTDs, there were reports in the included literature of men abusing their power by obstructing their female partner’s access to healthcare and necessities such as bandages, ointment and food [23]. This perhaps explains why some women opted to continue working, to the detriment of their health, and builds upon previous studies in which women with NTDs have been found to be at increased risk of adverse health-related outcomes compared to their male counterparts [17,64]. Indeed, the 2030 Agenda for Sustainable Development highlights the importance of gender roles and relations on health outcomes, with women recognised to be more at risk of inadequate access to healthcare [65].

According to Boukthir *et al*. [42] women were more affected than men with regard to the psychosocial implications of skin lesions. This is concordant with observations made in previous studies on SNTDs and gender, which concluded that women are disproportionately liable to adverse SNTD-related consequences [66]. Our research also shows that both males and females with SNTDs struggled to find a romantic partner, with women being more affected than men. This is consistent with existing literature that stresses that women are more liable to NTD-related social risk and discrimination [67]. Our research also indicates that social participation of women with SNTDs was more limited than that of their male counterparts [42,49,56]. Three female participants with LF in an Ethiopian study for instance felt they had no opportunities—a sentiment which was not expressed by any of study’s male participants [49]. This is linked to the strong influence of gender on social status and to the traditional role of men as breadwinner in the endemic regions, with female sex noted to be a risk factor for stigmatisation [68]. The concept of “triple jeopardy” has been described in the literature, whereby affected women’s gender, disabilities and disease-related stigma all compound the risk of discrimination [62,63]. Our research adds that this risk of discrimination is deeply influenced by expected societal roles for men and women.

Our study indicates that mental wellbeing was linked to disease knowledge, acceptance, and social support. The perception that a disease was curable and non-contagious also influenced disease acceptance and reduced the negative psychosocial impacts of SNTDs [40]. As noted, those study participants who were acceptant of their condition were primarily men who had experienced little or no stigmatisation and male and female individuals who had been reassured by their doctors [37,47,56,57]. This highlights the importance of disease knowledge, but also of the disparity in the lived experiences of men and women with SNTDs—emphasising the importance of conducting gender analysis when considering SNTDs with comorbid mental health issues [69]. An UNDP report exploring the gender dimensions of NTDs concludes that additional support must be made available to women and girls, who often require additional assistance to overcome social issues such as stigmatisation and lack of financial autonomy [70]. Successful peer support, and non-contributory social protection initiatives might have the secondary benefit of promoting financial independence and autonomy, conferring greater opportunities to women who may otherwise be dependent on abusive partners or family members [23].

Whilst data suggests that relatives’ violence towards women with SNTDs takes place across WHO regions, violence against men with SNTDs was more frequently reported in the African WHO region. Our review has identified commonalities across SNTDs, and we also recognise that the psychosocial impacts of SNTDs are heavily influenced by an individual’s immediate socio-economic environment [11]. More research is necessary to fully understand the precise drivers of violence towards men and women with SNTDs in the contexts in which these occur in order to inform appropriate strategies for prevention and control.

Across WHO regions, genders and SNTDs, common emotional impacts of SNTDs included low mood, anxiety and embarrassment. Self-stigmatisation was also common, largely attributed to fear of transmitting the infection, of the anticipated and actual socio-economic harms, and the perceived association between the SNTD and sinfulness. Stigmatisation was frequently cited as the biggest source of emotional distress [44,46,49,56], and is known to negatively influence health-seeking behaviours and treatment adherence [61,71]. Examples of enacted stigma ranged from unwanted attention, gossips, avoidance and exclusion to overtly hostile acts. Discrimination also occurred at an institutional level, with reported instances of men and women with SNTDs being barred from businesses, workplaces, public transport and even healthcare facilities. In turn the lived experiences of discrimination and self-stigmatisation commonly led to anticipated stigma and to self-stigmatisation, whereby individuals mistrusted society, with both men and women often opting to socially withdraw to minimise the risk of adverse reactions [44,46,49]. Importantly, our research suggests that aesthetic stigma may be a particular form of stigma affecting individuals with SNTDS. We have also shown that men’s fear of stigma often prompted them to seek early treatment, but women typically enjoyed less freedom to do so.

Some of the stigma related to SNTDs is also due to community misunderstandings around how NTDs are acquired [60]. In addition, lack of information, incomplete information, and inconsistent messages from health professionals about disease prognosis generated anxiety and despair among various informants [51]. Across genders, WHO regions and SNTDs, disease knowledge, early treatment, social support, and social acceptance were cited as protective factors, which helped prevent or ameliorate the damaging psychosocial consequences of SNTDs. This suggests that potential solutions may lie in culturally sensitive evidence-based education campaigns to promote the understanding of SNTDs among community members and healthcare professionals [17], coupled with initiatives that facilitate the integration and social participation of affected individuals, and with social care and support programmes that prevent and mitigate the financial and family burdens SNTDs pose on affected men and women and their relatives. Radio-based educational campaigns for instance have been widely reported to be affordable and effective at improving knowledge and reducing stigma [61,72,73]. Group cognitive behaviour therapy and contact-based educational campaigns where individuals with experiential knowledge tell their stories are also known to prevent and mitigate various forms of stigma and positively influence gender relations [74,75]. Finally, health workers SNTD training and social health protection initiatives are also necessary to prevent and mitigate the various psychosocial and financial strains, abuses and neglects that this paper shows, often arise alongside disabling and stigmatising SNTDs [23,76].

## Study limitations

This study is subject to a number of limitations. Firstly, due to the authors’ linguistic limitations articles included in this review are restricted to those published in the English language, potentially reducing the breadth of data analysed in this review. Additionally, most studies featured in this review present the results of interviews, translated to English. This renders the evidence vulnerable to potential inaccuracy, with loss or alteration of original meanings reported to be a problem [67].

The conclusions and findings of systematic reviews are contingent on the data reported in each included primary study, and there is a paucity of evidence pertaining to the experiences of individuals with most SNTDS and in most endemic contexts. Only eight SNTDs as experienced in nineteen countries are represented in this review, with seventeen (63%) studies focusing on leprosy and CL. Moreover, although SNTDs are united by common features, such as their visible manifestations and associated psychosocial risk, they are a highly heterogenous set of illnesses with a broad spectrum of manifestations, experienced in different ways by men and women of various ages living in socio-economically and politically different contexts and having themselves different degrees of assets and deprivations. This calls into question the generalisability of findings to other SNTDs and contexts. Despite these limitations, this review has identified important knowledge and public health action gaps that may help to improve the design and delivery of SNTD interventions. Qualitative research investigating the experiences of individuals with the remaining SNTDs and endemic contexts is still required to determine the full extent and nature of psychosocial implications.

With the available literature we are not able to consider variables which may affect and intersect with the reported lived experiences of SNTDs, beyond gender. For example, the socio-political and economic characteristics of the particular contexts, type of SNTD, and age of participants are not considered in the analysis. More research exploring the gendered psychosocial impacts of the different SNTDs in their various contexts is necessary.

## Conclusions

The psychosocial burden of SNTDs is a significant public health concern that requires a comprehensive approach addressing the illness’s physical, psychological, and socio-economic aspects. Although SNTDs have different aetiologies and transmission routes, they share symptoms, impacts, and a need for multi-dimensional management. Our review illustrates the wide-ranging and varied impacts of SNTDs on the socio-economic, psychological and physical wellbeing of affected men and women and some of the complex pathways of influence. We show how the psychosocial effects of SNTDs are highly gendered, largely owing to disparities in the social status, gender roles, and perceived value of affected men and women in endemic contexts. As shown, the psychosocial impacts are further compounded by poverty and limited economic opportunities, insufficient health care access, and scarce social protection schemes. Differences and commonalities within and between genders and settings were identified. Across genders, contexts, and SNTDs, ensuring healthcare access and prompt treatment, developing non-contributory social protection initiatives, and increasing disease knowledge among community members and healthcare professionals are all crucial to preventing and mitigating the negative psychosocial impacts of SNTDs. This said, more research is needed to effectively strategise how best to implement these in the different contexts in which SNTDs and gender collide to shape specific psychosocial impacts.

## Supporting information

S1 Table Quality Checking of included studies. (DOCX)

S2 Table Data from the included studies, disaggregated by gender, SNTD type, and endemic country. (XLSX)
