# Correction: The psychosocial impacts of skin-neglected tropical diseases (SNTDs) as perceived by the affected persons: A systematic review

**DOI:** 10.1371/journal.pntd.0014781

**Published:** 2026-10-06

**Authors:** Dasha L. Alderton, Caroline Ackley, Mei L. Trueba

The affiliation for the authors is incorrect. The correct affiliation is: Department of Global Health and Infection, Brighton and Sussex Medical School, University of Brighton and University of Sussex, Brighton, BN1 9PX.
