# Supplementary material for: The psychosocial impacts of skin-neglected tropical diseases (SNTDs) as perceived by the affected persons: A systematic review
Source: PLoS Negl Trop Dis. 2024 Aug 2;18(8):e0012391. doi: 10.1371/journal.pntd.0012391 (PMC11324132; doi:10.1371/journal.pntd.0012391)
Supplement: S1 Table — (DOCX) [file pntd.0012391.s001.docx]

**S1 Table.** Quality Checking
